# Hospital Sunday

**Published:** 1891-05-30

**Authors:** 


### Passing Topics.

### HOSPITAL SUNDAY.

The annual festival of St. Hospital appropriately marks the zenith of the charitable year. By the time the congregations are invited to help forward what is incomparably the greatest, most philanthropic, and most far-reaching of our charitable undertakings, the various and varied efforts which mark the season from Christmas to June have mostly culminated, and when the sun is high in the heavens, and the warmth of summer is in the air, Hospital Sunday comes, bearing the tribute religion offers to humanity.

It is fitting the offering should be made at this time, while yet the evidences of secular energy are fresh, because it emphasises the fact, sometimes in danger of being overlooked, that Hospital Sunday is not intended to supplant, but to supplement the ordinary methods of appeal. Time was, no doubt, when an over-sanguine estimate was formed of the result of the annual collection, and we can well remember how at the time of the establishment of the Fund, certain journals which ought to have been better informed, ventured upon prophecies, the falsification of which followed as a matter of course. But notwithstanding Hospital Sunday has failed to satisfy even less ambitious hopes than these, and although it is idle to deny that the total collection falls far short of what might be reasonably looked for from the flower of the mighty population of London, yet the institution is a great and, we hope, a growing one—great, not more, perhaps, in its present and tangible result, attained at a satisfactorily moderate cost, than in that subtle power and influence, whose best evidences may be reserved for a future day.

Certain it is that the more we can induce congregations to recognise the religious value of good deeds, and to ally practical benevolence with piety, the better for such institutions as our hospitals. It has been akin to a reproach that while millions of money are raised annually in the metropolis for missionary and other purely religious societies, whose chief work, moreover, is often carried on beyond the seas, the organizations which have for their object the relief of bodily pain and the mitigation of bodily infirmity should have commanded so little substantial sympathy on the part of the church-going population.

We might urge as a reason for the congregational support of our hospitals, the community of suffering by rich and poor alike, and the consequent community of interest both classes have in the maintenance of those temples where the gospel of healing is taught and practised; or, again, we might plead for our hospitals as powerful auxiliaries to missionary effort at home. Not long ago a letter came into our hands from the wife of a poor man, who, after being discharged as incurable from one of the London hospitals, went away to die in his cottage home, situated in a distant country village. One of his last-expressed wishes was that the chaplain of that hospital should know it was to his ministrations the dying man owed all he had of the hope which animated him at that supreme moment. In this and the many other instances which the work of every hospital affords, we may claim with confidence to discern such evidence of the religious value of our hospitals as should obtain for them a generous support even from those professors of religion whose warmest sympathies are not so freely given as they might be to the work of healing and assuaging bodily suffering.

Agricola.

